# Mechanistic insights into SARS-CoV-2 spike protein induction of the chemokine CXCL10

**DOI:** 10.1038/s41598-024-61906-6

**Published:** 2024-05-16

**Authors:** Davoud Ghazanfari, Maria Cecilia Courreges, Lydia E. Belinski, Michael J. Hogrell, Jacob Lloyd, Stephen C. Bergmeier, Kelly D. McCall, Douglas J. Goetz

**Affiliations:** 1 Department of Chemical and Biomolecular Engineering, Ohio University, Athens, OH 45701 USA; 2 Department of Specialty Medicine, Ohio University, Athens, OH 45701 USA; 3 Biomedical Engineering Program, Ohio University, Athens, OH 45701 USA; 4 Department of Chemistry and Biochemistry, Ohio University, Athens, OH 45701 USA; 5 The Diabetes Institute, Ohio University, Athens, OH 45701 USA; 6 Molecular and Cellular Biology Program, Ohio University College of Arts & Sciences, Athens, OH 45701 USA; 7 Department of Biological Sciences, Ohio University College of Arts & Sciences, Athens, OH 45701 USA; 8 Department of Biomedical Sciences, Ohio University Heritage College of Osteopathic Medicine, Athens, OH 45701 USA

**Keywords:** SARS-CoV-2-Spike Protein, CXCL10 (IP-10), Glycogen Synthase Kinase-3, TLR2, IRF, NF-κB, Biomedical engineering, Viral infection, Target identification

## Abstract

During a SARS-CoV-2 infection, macrophages recognize viral components resulting in cytokine production. While this response fuels virus elimination, overexpression of cytokines can lead to severe COVID-19. Previous studies suggest that the spike protein (S) of SARS-CoV-2 can elicit cytokine production via the transcription factor NF-κB and the toll-like receptors (TLRs). In this study, we found that: (i) S and the S2 subunit induce *CXCL10*, a chemokine implicated in severe COVID-19, gene expression by human macrophage cells (THP-1); (ii) a glycogen synthase kinase-3 inhibitor attenuates this induction; (iii) S and S2 do not activate NF-κB but do activate the transcription factor IRF; (iv) S and S2 do not require TLR2 to elicit CXCL10 production or activate IRF; and (v) S and S2 elicit CXCL10 production by peripheral blood mononuclear cells (PBMCs). We also discovered that the cellular response, or lack thereof, to S and S2 is a function of the recombinant S and S2 used. While such a finding raises the possibility of confounding LPS contamination, we offer evidence that potential contaminating LPS does not underly induced increases in CXCL10. Combined, these results provide insights into the complex immune response to SARS-CoV-2 and suggest possible therapeutic targets for severe COVID-19.

## Introduction

Often, one of the key features of severe COVID-19 is a “cytokine storm”, i.e., the presence of unwanted and unneeded cytokines^1–3^. A dysregulated innate immune response, in reaction to pathogen invasion, underlies cytokine storms^3,4^. Innate immune cells, such as macrophages and dendritic cells located in the tissues, sense pathogen associated molecular patterns (PAMPs) via pattern recognition receptors (PRRs) such as toll-like receptors (TLRs)^4–6^. Ligated PRRs trigger signaling pathways which activate transcription factors such as NF-κB, and interferon regulatory factors (IRFs)^4,7,8^. Transcription factor activation leads to the release of cytokines and chemokines (a subset of cytokines) which is advantageous since their expression facilitates the elimination of the invading pathogen^4^. However, a hyper-response can culminate in a cytokine storm that can lead to tissue damage, organ failure, and death^9,10^.

Several lines of evidence suggest that the spike protein from SARS-CoV-2 can induce cytokine expression^2,11–16^. In addition to the spike protein (S), which has two main subunits, S1 and S2, the SARS-CoV-2 virus has three other structural proteins: nucleocapsid (N), membrane (M) and envelope (E)^17,18^. Khan et al. treated human macrophage THP-1 cells and peripheral blood mononuclear cells (PBMCs) with S, M, N, and E proteins and found that only the S protein induced cytokine expression^13^. Further, they reported that TLR2, but not TLR4, recognizes trimeric S protein and the S2 subunit inducing the gene expression of *Il-6*, *Il-1β*, and *Tnfα* via a MyD88-dependent NF-κB signaling pathway^13^. In contrast, Zheng et al. reported that the E, but not the S, protein induced cytokine gene expression in bone marrow-derived macrophages (BMDMs)^16^. Zhao et al. found that purified spike trimer, but not the receptor binding domain (RBD) nor the N protein, induces the expression of the *IL-1β* gene by THP-1 macrophages^14^. In contrast to Khan et al.’s finding regarding the importance of TLR2, but not TLR4^13^, Zhao et al. found evidence for the involvement of TLR4 but not TLR2 and TLR3^14^. Pantazi et al. found that the S protein induces the expression of *IL-6*, *MIP1a* and *TNFα* by THP-1 macrophages^15^. Further, they investigated the effect of the S protein on LPS- and Pam3CSK4-activated macrophages and PBMCs and found that the S protein augmented the expression of LPS-induced *IL-6* gene expression^15^.

Combined, an emerging hypothesis, supported by some but not all^19,20^ of the work in this area, is that the spike protein itself induces cytokine expression and this induction may contribute to SARS-CoV-2 cytokine storms. While many molecules and pathways have been implicated in the induction of the cytokine storms [e.g. the TLR family (specifically TLR2 and TLR4), the NF-κB pathway], the mechanism by which the spike protein induces cytokine expression clearly remains unresolved.

We recently found evidence that the S and S2 subunit of SARS-CoV-2 induce production of the CXCL10 protein, a chemokine that has been found to be elevated in severe COVID-19^12^. Further, we found that a highly selective inhibitor of GSK-3^21^, a kinase implicated in PAMP-induced cytokine expression^22,23^, attenuated this induction. However, the mechanism by which the induction occurs was largely unexplored. This fact, combined with the discussion presented above, led us to further probe SARS-CoV-2 spike protein induction of CXCL10.

## Results

### COB-187 significantly reduces AS- and AS2-induced CXCL10 protein levels as revealed by a proteome profiler human cytokine array

**Figure 1:**
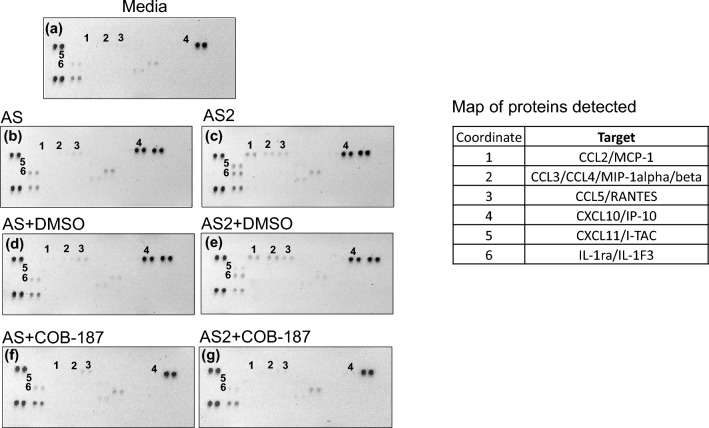
AS and AS2 treatment increase CXCL10 protein levels and this increase is attenuated by COB-187. Protein arrays exposed to supernatants from THP-1 macrophages treated with AS and AS2 proteins + /− COB-187 revealed: supernatants from untreated THP-1 macrophages had little, if any, CXCL10 (location 4, panel **a**); AS or AS2 protein alone and in the presence of 1% DMSO (carrier control for COB-187) increased CXCL10 protein levels (location 4 panels **b**–**e**); COB-187 appeared to abolish the AS- and AS2-induced increases in CXCL10 protein levels (location 4 panels **f**, **g**). Note that AS2 appeared to increase the levels of 3 other proteins (locations 1,2,3; panels **c**) that were dramatically reduced by COB-187 (locations 1,2,3; panel **g**) but unaffected by 1% DMSO (locations 1,2,3; panel **e**). The map provides the correspondence between the number on the array and the cytokine present at that location. Each cytokine is in duplicate (two dots per location). The two dark pairs of dots on the left side of each array and the dark pair of dots on the right side of the array are positive controls. Results shown are from a single experiment. The results from a replicate experiment are provided in Supplementary Fig. S1.

In our previous publication, we reported that S and S2 proteins induce robust increases in CXCL10 protein levels by THP-1 macrophages as determined by an enzyme-linked immunoassay (ELISA)^12^. (The source of the recombinant spike proteins was ACROBiosystems. We refer to these as AS and AS2 from this point forward to distinguish them from S2 proteins obtained from other sources used later in the study.) This increase in CXCL10 protein levels was dramatically attenuated by select clinically relevant GSK-3 inhibitors and COB-187 (a specific GSK-3 inhibitor^21^)^12^. In the present study, we sought to probe, via an alternate assay, the effect of AS and AS2 on CXCL10 protein levels and the ability of COB-187 to attenuate any observed increases. Thus, a proteome profiler human cytokine array that allows the simultaneous detection of 36 distinct cytokines/chemokines was exposed to supernatants from THP-1 macrophages treated with AS and AS2 proteins in the presence or absence of COB-187 (Fig. 1).

The blots exposed to supernatants from AS and AS2 treated-THP-1 macrophages contained one pair of distinct dots (location 4, Fig. 1b, c, respectively) that were not present in the blot exposed to supernatants from control THP-1 macrophages (media control; location 4, Fig. 1a). The dots at location 4 are at the location of capture antibodies for CXCL10. Further, the pixel density of the CXCL10 dots appeared to remain constant when THP-1 cells were treated with AS and AS2 proteins in the presence of 1% DMSO (carrier control for COB-187) (location 4, Fig. 1d, e, respectively), while the density was reduced to background levels in the presence of 25 µM COB-187 (location 4, Fig. 1f,g). Thus, in line with our previously published results^12^, AS and AS2 appear to increase CXCL10 protein levels in THP-1 macrophages and this increase is attenuated by COB-187 (Fig. 1).

As noted above, the array used in this study has antibodies for 36 distinct cytokines/chemokines. We have not completed a full analysis of the effect of AS and AS2 on all of these cytokines, but a couple of findings are consistent between the two replicate experiments (Figs. 1 and Supplementary Fig. S1). Most notably, AS2 appeared to consistently increase CCL2, CCL3/CCL4, and CCL5 protein levels (locations 1, 2, 3, Fig. 1c) above background levels and each of these increases were attenuated by COB-187 (locations 1, 2, 3 Fig. 1g); 1% DMSO did not appear to affect these increases (locations 1, 2, 3 Fig. 1e). Note that the dots marked as 5 and 6 did seem to increase with AS2 treatment but this increase appeared to be diminished by 1% DMSO (Fig. 1c,e).

Given that an ELISA^12^ and the current array assay (Fig. 1) revealed consistent and robust AS and AS2-induced increases in CXCL10 protein levels, we focused on AS and AS2 effects on CXCL10 for the remainder of the present study.

### AS and AS2 induce gene expression of *CXCL10* and COB-187 significantly reduces this induction

**Figure 2:**
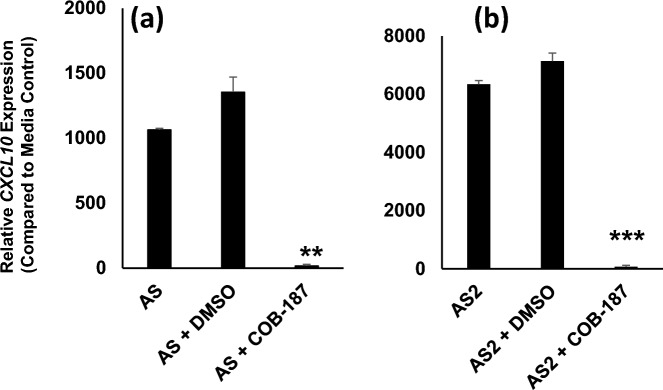
AS and AS2 induce *CXCL10* gene expression and COB-187 significantly reduces this induction. THP-1 macrophages were treated with AS (**a**) or AS2 (**b**) for 6 h. Subsequently, RNA was harvested and evaluated via RT-qPCR. The AS and AS2 proteins dramatically induced *CXCL10* expression. COB-187 significantly reduced the induction while 1% DMSO (carrier control for COB-187) did not reduce the induction. The y-axis represents the *CXCL10* expression normalized to the media control. Error bars: SD generated from two separate experiments. **P < 0.01 and ***P < 0.001 were determined by a *t*-test comparing the *CXCL10* expression ratio for the COB-187 condition to the DMSO condition in a given panel. AS, AS2 concentration, 25 nM; COB-187 concentration, 25 μM.

To gain insight into the mechanism of AS- and AS2-induced increases in CXCL10 protein levels and COB-187 inhibition of this induction, the effect of AS and AS2 on THP-1 macrophage *CXCL10* gene expression was evaluated. Specifically, THP-1 macrophages were treated with AS and AS2 in the presence or absence of COB-187 or 1% DMSO (carrier control) for 6 h. The macrophages were subsequently harvested for RNA isolation and RT-qPCR analysis which revealed that AS and AS2 dramatically induced *CXCL10* gene expression (Fig. 2a,b). Interestingly, the AS2 induction of *CXCL10* was ~ six-fold higher than AS induction. The AS and AS2 induction of *CXCL10* was significantly and dramatically reduced by co-treatment with COB-187 but not reduced by DMSO (Fig. 2a,b). Note that COB-187 had little, if any effect, on the expression of the housekeeping genes (data not shown) indicating that COB-187’s inhibitory effect on *CXCL10* expression is specific. These observations are in line with our findings at the protein level [^12^ and Fig. 1] and suggest that the mechanism of AS and AS2 induction of CXCL10 protein levels is at the transcriptional level.

### *TLR2* mRNA is robustly expressed and TLR2 protein is present on THP-1 macrophages

**Figure 3:**
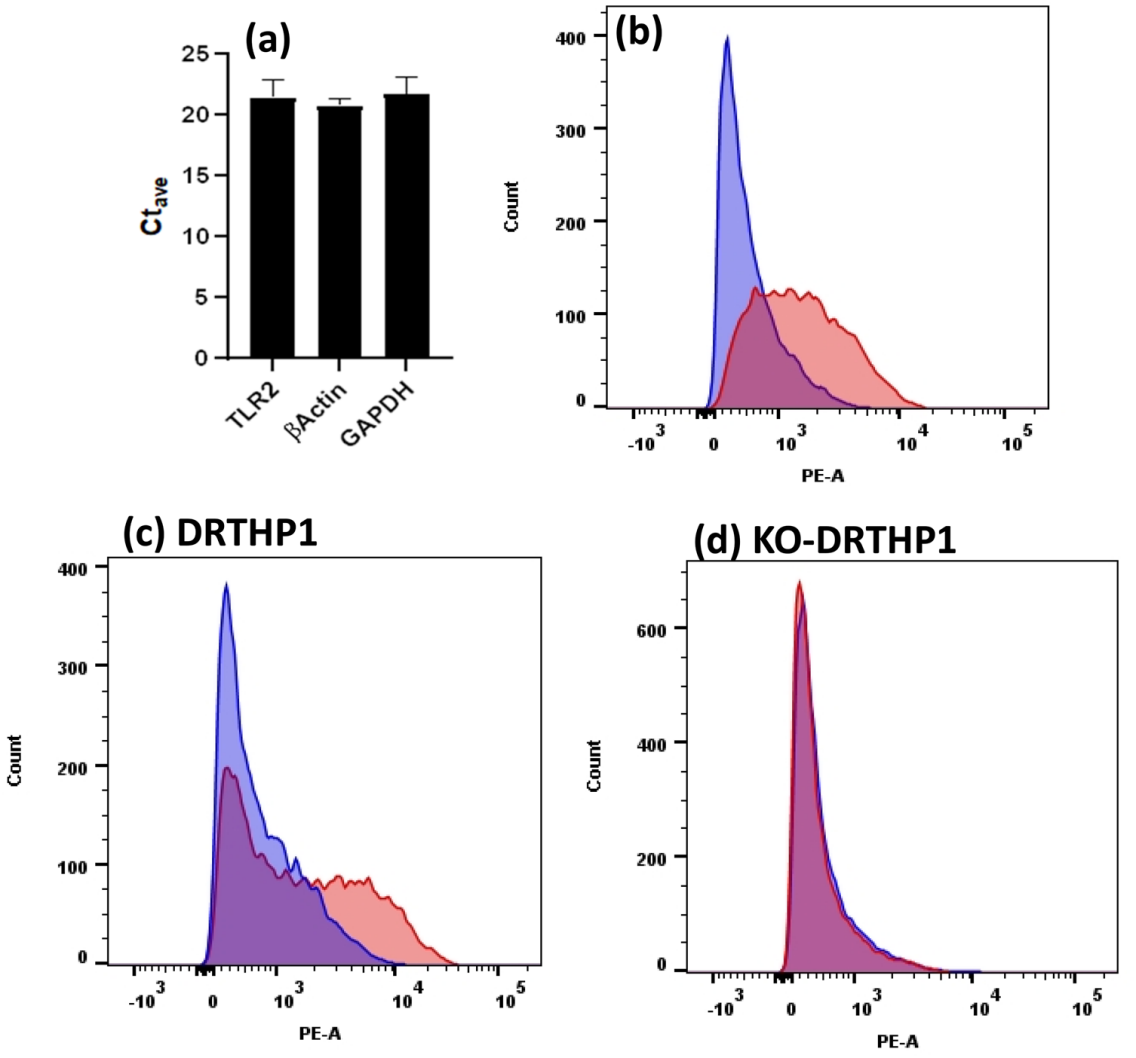
TLR2 expression, or lack thereof, by THP-1, DRTHP1 and KO-DRTHP1 macrophages. (**a**) The level of expression of *TLR2*, *βActin*, and *GAPDH* was assessed using RT-qPCR. The y-axis represents the cycle threshold (Ct) [the number of cycles required to generate fluorescence signal greater than background signal (the minimum fluorescence signal that the PCR machine can detect)]. The obtained values indicate the abundant expression of each gene. Error bars represent SD generated from three separate experiments. (**b**, **c**, **d**) Flow cytometric analysis revealed that a mAb to TLR2 bound to THP1 (**b**), DRTHP1 (**c**), but not the KO-DRTHP1 (**d**) macrophages (red histograms) to a greater extent than an isotype matched control (blue histograms) suggesting that the THP-1, DRTHP1, but not the KO-DRTHP1 macrophages, express surface TLR2 protein. Results from representative experiments are shown for panels (**b**, **c**, **d**). The additional replicates are presented in Supplementary Fig. S2.

Others have reported that SARS-CoV-2 spike proteins induce cytokines and chemokines via TLR2 and the NF-κB signaling pathways^13,16^. Further, Zhao et al. reported that the S protein induces cytokines via a TLR4-dependent pathway^14^. We recently found that LPS-RS, an antagonist of TLR4, significantly inhibits LPS-induced increases in CXCL10 protein levels while having little, if any, effect on AS- and AS2-induced increases in CXCL10 protein levels^12^. These findings suggest that AS and AS2 do not require TLR4 to induce increases in CXCL10 protein levels^12^. Thus, we investigated a potential role for TLR2 in AS- and AS2-induced increases in CXCL10. Specifically, we first determined that THP-1 macrophages express *TLR2* (Fig. 3a) and that the TLR2 protein is present on the cell surface (Fig. 3b). Hence, THP-1 macrophages appear to express TLR2 and it is possible that TLR2 is involved in AS- and AS2- induced increases in CXCL10.

### AS and AS2 increase CXCL10 protein levels by THP-1 macrophages lacking TLR2

To probe the potential role of TLR2 in AS- and AS2-induced increases in CXCL10 protein levels, we decided to make use of THP-1 *TLR2* knockout cells. Specifically, we obtained dual reporter THP-1 cells (DRTHP1) and a *TLR2* knockout version of these cells (KO-DRTHP1). The DRTHP1 cells report NF-κB and IRF activation (further described below). Thus, the choice of using the DRTHP1 and KO-DRTHP1 cells not only allowed us to investigate a possible contribution of TLR2, but also possible involvement of NF-κB and IRF; two transcription factors that play a key role in PAMP-induced cytokine expression^24,25^.

**Figure 4:**
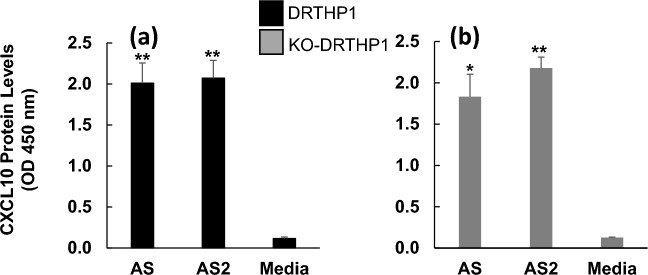
AS and AS2 treatment increase CXCL10 protein generation by THP-1 macrophages lacking *TLR2*. DRTHP1 (panel **a**) and KO-DRTHP1 (panel **b**) macrophages were treated with AS and AS2 proteins for 6 h and the levels of CXCL10 protein in the supernatants were subsequently determined. The y-axis represents AS- or AS2-induced CXCL10 protein levels as indicated by OD 450 nm. The black and grey bars represent DRTHP1 and KO-DRTHP1 macrophages, respectively. Error bars represent SD generated from duplicates for the representative experiment shown. *P < 0.025 and **P < 0.01 as determined by a *t*-test comparing the AS and AS2 condition to media control. Results from other replicates and further statistical details are presented in Supplementary Table S1. AS, AS2 concentration, 25 nM. The concentration of CXCL10 in the supernatants for the AS and AS2 conditions were ~ 4000 pg/ml.

We first utilized flow cytometric analysis to evaluate DRTHP1 and KO-DRTHP1 macrophage levels of TLR2. This analysis revealed that, similar to the wild type THP-1 macrophages (Fig. 3b), DRTHP1 macrophages appeared to present TLR2 (Fig. 3c). In contrast, and as expected, TLR2 was not observed on KO-DRTHP1 macrophages (Fig. 3d). Both DRTHP1 and KO-DRTHP1 macrophages treated with AS and AS2 had significantly elevated levels of CXCL10 protein in their supernatants (Fig. 4). Further, CXCL10 protein levels appeared similar between the two cell lines (Fig. 4). These results suggest that TLR2 is not required for AS- and AS2-induced increases in CXCL10.

### AS and AS2 do not appear to activate NF-κB but do activate IRF, and TLR2 is not required for IRF activation

The THP-1 dual reporter cells (DRTHP1 and KO-DRTHP1) contain embryonic alkaline phosphatase (SEAP) and Lucia luciferase reporter constructs. Upon activation of NF-κB or IRF, respectively, the cells produce the SEAP and Lucia reporter proteins which are secreted into the supernatant. Thus, the level of SEAP and Lucia proteins in the supernatants, determined via QUANTI Blue and QUANTI Luc assays, respectively, correlate with the activation of NF-κB and IRF. We used these cells to determine the effect of AS and AS2 on NF-κB and IRF activation.

**Figure 5:**
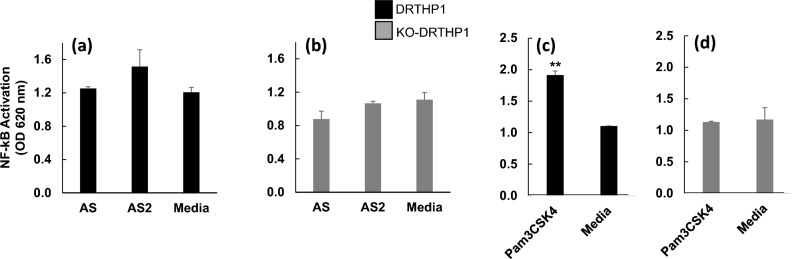
AS and AS2 do not appear to activate NF-κB in DRTHP1 and KO-DRTHP1 macrophages. DRTHP1 and KO-DRTHP1 macrophages were treated with AS, AS2, or Pam3CSK4 for 6 h. Subsequently, the supernatants were harvested and analyzed using the QUANTI blue assay to determine the level of NF-κB activation. The y-axis represents NF-kB activation as indicated by OD 620 nm. The black and grey bars represent data generated using supernatants harvested from DRTHP1 and KO-DRTHP1 macrophages, respectively. AS and AS2 did not induce NF-κB activation in DRTHP1 (**a**) nor KO-DRTHP1 (**b**) macrophages to a level that was significantly higher than the media control. Pam3CSK4 induced significant NF-κB activation in DRTHP1 macrophages (**c**) but not in KO-DRTHP1 macrophages (**d**). Error bars represent SD generated from duplicates for the representative experiments shown. **P < 0.01 as determined by a *t*-test comparing the Pam3CSK4 treatment condition to media control. Results from additional replicates and further statistical details are presented in Supplementary Table S2. AS, AS2 concentration, 25 nM. Pam3CSK4 concentration, 1000 ng/mL.

**Figure 6:**
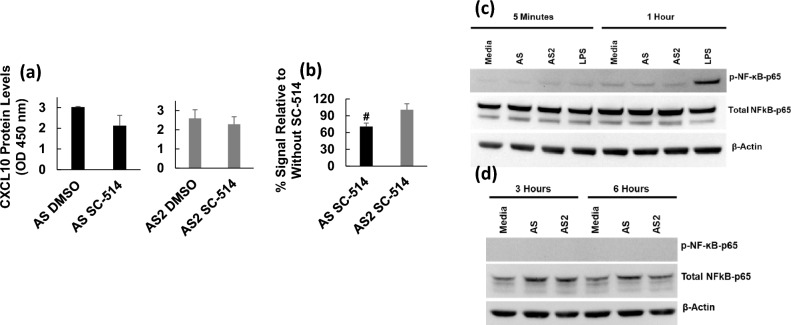
AS and AS2 do not appear to activate NF-κB in THP-1 macrophages. THP-1 macrophages were treated with AS or AS2 in the presence of SC-514 (100 μM) or carrier control (0.6% DMSO) for 6 h and the levels of CXCL10 protein in the supernatants were subsequently determined. The y-axis represents AS- or AS2-induced CXCL10 protein levels as indicated by OD 450 nm. The black and grey bars represent treatment with AS and AS2 spike proteins, respectively. (**a**) A representative result from 3 replicates is shown. (**b**) For each replicate, the OD level obtained from treatment with AS or AS2 and SC-514 was divided by the OD level obtained from treatment with AS or AS2 and carrier control (DMSO) and the resulting quotient multiplied by 100 to arrive at the % signal. The % signal for each of the replicates was averaged and the result presented. Error bars represent the SD generated from duplicates for the representative experiment shown in (**a**) and the SD generated from the average % signal of the 3 replicates shown in (**b**). #*P* < 0.05 as determined by a one sample *t*-test comparing the average to 100%. Results from other replicates and further statistical details are presented in Supplementary Table S3. AS, AS2 concentration, 25 nM. The concentration of CXCL10 in the supernatants for the AS and AS2 condition in the presence of DMSO was ~ 3000 pg/ml. (**c** and **d**) Total protein isolated from THP-1 macrophages at the time points indicated were subjected to SDS-PAGE and western blot analysis. AS and AS2 did not appear to induce increased phospho-NF-κB p65 at any of the time points investigated (**c** and **d**), while LPS did (see 1 h time point in **c**). AS and AS2 concentration, 25 nM. Note that (**c**) and (**d**) are from separate experiments and original blots are shown in Supplementary Fig. S3.

**Figure 7:**
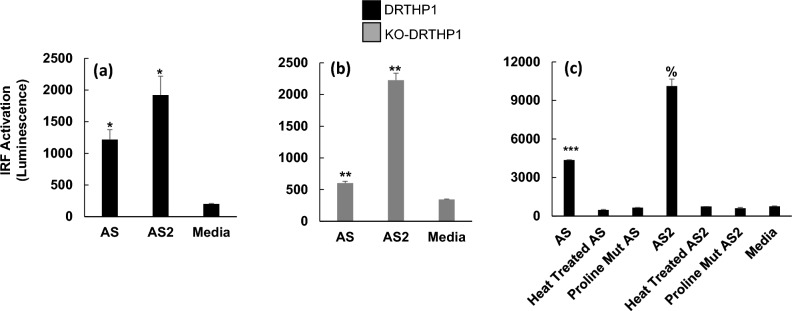
AS and AS2 induce IRF activation in DRTHP1 and KO-DRTHP1 macrophages. DRTHP1 and KO-DRTHP1 macrophages were treated with AS, AS2, heat-treated AS, heat-treated AS2, mutated AS, or mutated AS2 for 6 h. Subsequently, the supernatants were harvested and analyzed using the QUANTI Luc assay to determine the level of IRF activation. The y-axis represents IRF activation as indicated by luminescence. The black and grey bars represent data generated using supernatants harvested from DRTHP1 and KO-DRTHP1 macrophages, respectively. AS and AS2 induced IRF activation in DRTHP1 (**a**) and KO-DRTHP1 (**b**) macrophages to a level that was significantly higher than the media control. (**c**) DRTHP1 macrophages were treated with wild type AS or AS2 that were not, or were, heat-treated prior to use in the assay or proline mutated versions of AS and AS2 for 6 h and the level of IRF activation subsequently determined. Error bars represent SD generated from duplicates for the representative experiments shown. *P < 0.025, **P < 0.01, %*P* < 0.0083 (the cutoff for significance with a Bonferroni correction involving 6 comparisons) and ***P < 0.001 as determined by a *t*-test comparing the treatment condition to media control. Results from additional replicates and further statistical details are presented in Supplementary Table S4. AS, AS2 concentration, 25 nM.

DRTHP1 and KO-DRTHP1 macrophages were treated with AS and AS2 for 6 h. Subsequently the supernatants were harvested and analyzed by the QUANTI Blue (to detect NF-κB activation Fig. 5), and the QUANTI Luc (to detect IRF activation Fig. 7) assays. Neither AS nor AS2 appeared to induce NF-κB activation significantly above background levels for either the DRTHP1 or KO-DRTHP1 macrophages (Fig. 5a,b). In contrast, Pam3CSK4 [a TLR2 agonist that has been reported to induce NF-κB activation^26^] induced NF-κB activation in DRTHP1 macrophages (Fig. 5c), and this activation was attenuated by loss of *TLR2* (Fig. 5d). While the data in Fig. 5 suggests that neither AS nor AS2 activate NF-κB, there was a high background signal present in these cells (e.g. Fig. 5a, third bar). Thus, we probed the question of activation of NF-κB using a NF-κB activation inhibitor, specifically SC-514, and western blotting. We treated THP-1 macrophages with AS and AS2 in the presence of SC-514. In replicate experiments, SC-514 did not cause a significant decrease in the level of CXCL10 protein detected post-treatment with AS or AS2 (Fig. 6a; Supplementary Table S3 yellow block of data); albeit when the AS data was averaged together over multiple experiments, it did appear that there was a modest, ~ 30%, reduction (Fig. 6b; black bar) but this was not seen for AS2 (Fig. 6b; grey bar). In addition, western blot analysis revealed no clear increase in phosphorylated NF-κB p65 at multiple time points (1, 3 and 6 h) upon treatment with AS and AS2 while LPS, used as a positive control, did cause an increase (Fig. 6c,d). These findings do not rule out involvement of NF-κB (e.g. there could be some basal level of nuclear NF-κB present in the differentiated THP-1 cells). That said, the combined data suggests a limited, if any, role for AS and AS2 induced activation of NF-κB in the mechanism of CXCL10 induction.

In contrast to the lack of induction of NF-κB, both AS and AS2 induced significant, compared to media control, IRF activation in DRTHP1 macrophages (Fig. 7a; and Supplementary Table S4 yellow block of data). Further, both AS and AS2 induced significant activation of IRF in the KO-DRTHP1 macrophages in two of the three replicate experiments and appeared to increase activation, albeit not to a significant level, in the other replicate (Figs. 7b and Supplementary Table S4 orange block of data) suggesting that AS and AS2 can activate IRF through a TLR2-independent mechanism. Drawing conclusions regarding whether there is a TLR2-*dependent* mechanism of AS and AS2 activation of IRF is not straight-forward. Specifically, the data in Fig. 7 indicates that AS-induced IRF activation is lower for the KO-DRTHP1 macrophages relative to the DRTHP1 macrophages, suggesting a TLR2-dependent mechanism. That said, when all of the replicates are considered, (Supplementary Table S4 yellow and orange blocks of data) it is not clear that such a conclusion can be drawn.

To further probe the specificity of the AS and AS2 induction of IRF, we heat-treated the AS and AS2 preparations prior to use in the activation assay. Heat-treated AS and AS2 proteins did not activate IRF in DRTHP1 macrophages while non-heat-treated AS and AS2 preparations did (Fig. 7c). We have previously reported that AS and AS2 proteins with proline substitutions [specifically: F817P, A892P, A899P, A942P, K986P, V987P] do not cause increased CXCL10 protein levels in THP-1 cells^12^. We tested the proline mutated proteins in the IRF induction assay and found that they did not appear to activate IRF (Fig. 7c).

Combined, the data in Figs. 5, 6 and 7 indicate that AS and AS2 do not activate NF-κB but do activate IRF.

### Treatment of PBMCs with AS and AS2 increases CXCL10 protein levels, while treatment with S2 from two other sources does not

**Figure 8:**
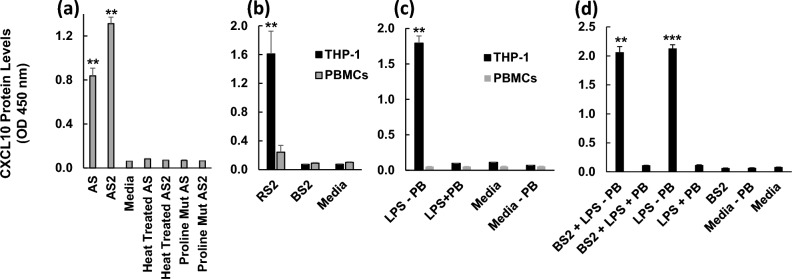
AS and AS2 treatment increase CXCL10 protein generation by PBMCs but RS2 and BS2 do not. (**a**) PBMCs were treated with AS, AS2, heat-treated AS, heat-treated AS2, mutated AS, mutated AS2 proteins, or media for 6 h. The concentration of CXCL10 in the supernatants for the AS and AS2 condition was ~ 2000 pg/ml and ~ 4000 pg/ml, respectively. **P < 0.01 as determined by a *t*-test comparing the treatment condition to media control. (**b**) THP-1 macrophages and PBMCs were treated with RS2, BS2, or media for 6 h. The concentration of CXCL10 in the supernatants for the RS2 THP-1 condition is ~ 4000 pg/ml. **P < 0.01 as determined by a *t*-test comparing the treatment condition to media matched control. (**c**) THP-1 and PBMCs were treated with LPS without PB, LPS in the presence of 30 μg/ml PB, media with 30 μg/ml PB (media), or media alone (media–PB). **P < 0.01 as determined by a *t*-test comparing the treatment condition to media matched control. (**d**) THP-1 macrophages were treated with BS2 preincubated with LPS in media without PB prior to addition to the cells, BS2 preincubated with LPS followed by addition of 30 μg/ml PB prior to addition to the cells, LPS in media without PB, LPS in media containing 30 μg/ml PB, BS2 in media without PB, media alone (media–PB), or media with 30 μg/ml PB (media). Concentration of LPS, 1 EU/ml. **P < 0.01 and ***P < 0.001 as determined by a *t*-test compared to same condition with PB. After treatment, the levels of CXCL10 protein in the supernatants were determined. The y-axis represents CXCL10 protein levels as indicated by OD 450 nm. The black and grey bars represent THP-1 macrophages and PBMCs, respectively. Error bars represent SD generated from duplicates for the representative experiment shown. Results from other replicates (**a**–**c**) and conditions (**d**), and further statistical details are presented in Supplementary Table S5. Spike protein concentration, 25 nM.

To determine if AS and AS2 could induce CXCL10 protein secretion from a more relevant cell type, we tested their effects on peripheral blood mononuclear cells (PBMCs). As shown in Fig. 8a, 6 hour treatment with either AS or AS2 significantly increased CXCL10 levels in the PBMC supernatants. Heat-treated AS and AS2 proteins and AS and AS2 proteins with proline substitutions [specifically: F817P, A892P, A899P, A942P, K986P, V987P] did not increase CXCL10 protein levels (Fig. 8a). Thus, it appears that AS and AS2 induce production of CXCL10 by PBMCs.

We decided to test the ability of S2 from another supplier, specifically RayBiotech (we term this RS2), to induce CXCL10 production by PBMCs to confirm the results with AS2. We used THP-1 macrophages as a positive control. As expected, RS2 induced CXCL10 production by THP-1 macrophages but, surprisingly, not by PBMCs (Fig. 8b). To further investigate this issue, we purchased S2 from Biotechne (we term this BS2) and found that BS2 did not induce CXCL10 production by either THP-1 macrophages or PBMCs (Fig. 8b).

Several factors could contribute to these inconsistencies including, as has been highlighted in a recent review^27^, the presence of contaminating LPS in the protein preparations. To investigate this possibility, we treated THP-1 macrophages and PBMCs with LPS and found that LPS induced CXCL10 production by THP-1 macrophages but not by PBMCs (Fig. 8c) indicating that potential contaminating LPS present in a protein preparation used to treat PBMCs would not induce CXCL10. It is important to note that all the spike protein solutions used to treat cells in the present study contained polymyxin B (PB) which has been shown to inhibit the activity of contaminating LPS in other protein preparations^28^ including the S1 spike protein^19^. Indeed, PB clearly ablates LPS induction of CXCL10 production as shown in Fig. 8c, where treatment of THP-1 macrophages with LPS in the presence of PB eliminates induced CXCL10 production, a finding we have reported previously^12^. Finally, we preincubated BS2 with LPS and treated THP-1 macrophages with this solution in the presence or absence of PB (Fig. 8d). The solution without PB induced CXCL10 production and PB readily inhibited this induction (Fig. 8d) suggesting that the activity of any contaminating LPS-spike protein adduct would be inhibited by PB.

## Discussion

Our previous publication utilizing a CXCL10 ELISA demonstrated that AS and AS2 proteins cause an increase in the production of CXCL10 protein by THP-1 macrophages^12^. In addition, we provided evidence that select clinically relevant GSK-3 inhibitors and COB-187 dramatically attenuate the AS- and AS2-induced increase in CXL10 protein levels^12^.

In the present study, we gained insight into the mechanism by which AS and AS2 may induce CXCL10 protein production. A proteome cytokine array revealed that CXCL10 protein levels were consistently and robustly increased by AS and AS2 (Fig. 1) and that COB-187 drastically reduced this induced increase (Fig. 1), corroborating our previous findings with the CXCL10 ELISA^12^. We have not done a detailed analysis of which cytokines, in addition to CXCL10, are induced by AS and AS2 proteins. Clearly other cytokines are induced as evidenced by the fact that the protein array detected other cytokines (namely CCL2/MCP-1, CCL3/CCL4/MIP-1alpha/beta, CCL5/RANTES) in the THP-1 supernatants treated with the AS2 protein (Fig. 1). The induction of all of these cytokines was attenuated by COB-187 (Fig. 1) but not 1% DMSO (carrier control). With regards to the possible induction of other cytokines, we note that, as suggested by the manufacturer, we diluted the supernatants prior to use in the assay. It is reasonable to speculate that additional cytokines would be identified if undiluted supernatants were used. The RT-qPCR analysis revealed that AS and AS2 proteins induce *CXCL10* gene expression and that COB-187 significantly reduces this induction (Fig. 2). Combined, all of these findings support the hypothesis that the S and S2 SARS-CoV-2 proteins induce *CXCL10* gene expression, and a consequent increase in CXCL10 protein levels, and that S and S2 induction of *CXCL10* involves GSK-3.

Previous studies have implicated TLRs in SARS-CoV-2 spike protein-induced cytokine expression, but there has been some discord among these studies. One study suggests TLR2 but not TLR4 involvement^13^, while another implicated TLR4 but not TLR2^14^. We previously revealed that LPS-RS, an antagonist of TLR4, has little, if any, effect on AS and AS2-induced increases in CXCL10 protein levels, suggesting a TLR4-independent pathway^12^. In the present study, we found that both a *TLR2* knockout THP-1 cell line, namely KO-DRTHP1, and its parent cell line, DRTHP1, treated with AS or AS2 express robust levels of CXCL10 protein (Fig. 4). This result supports the hypothesis that the S and S2 proteins can induce increased CXCL10 protein levels via a TLR2-independent mechanism.

The DRTHP1 and KO-DRTHP1 have reporter constructs for NF-κB and IRF which allowed us to explore the effects of AS and AS2 on the activation of these two important transcription factors. This analysis indicated that neither AS nor AS2 induce significant increases in NF-κB activation (Fig. 5a,b). It is important to note that InvivoGen has reported that PMA, used to differentiate the DRTHP1 and KO-DRTHP1 cells to macrophages, can activate the NF-κB transcription factor^29^ leading to “background” levels of SEAP reporter protein. We did observe a basal signal in the QUANTI Blue assay, and this phenomenon could mask AS and AS2 activation of NF-κB. That said, the TLR2 agonist Pam3CSK4 induced detectable NF-κB activation in DRTHP1 macrophages, but not in KO-DRTHP1 macrophages, (Fig. 5c,d) demonstrating that the detection system was intact and reasonably sensitive. Further, the NF-κB inhibitor SC-514 did not cause a dramatic drop in AS and AS2-induced CXCL10 expression and we did not find increased phosphorylated NF-kB p65 upon treatment with AS or AS2 (Fig. 6). Both observations are in line with the hypothesis that AS and AS2 induce CXCL10 expression independent of NF-κB activation, a finding somewhat in contrast to Kahn et al.’s observation that S and S2 induce the gene expression of *Il-6*, *Il-1β*, and *Tnfα* via a MyD88-dependent NF-κB signaling pathway^13^.

In contrast to the findings for NF-κB, the IRF analysis revealed that AS and AS2 significantly induced activation of IRF (Fig. 7a). The role of TLR2 in this induction was somewhat complicated. AS and AS2 appeared to elicit IRF activation in the KO-DRTHP1 macrophages suggesting the presence of a TLR2-independent mechanism (Fig. 7b). The data was inconsistent regarding whether the loss of TLR2 lessened AS and AS2 induction of IRF (Supplementary Table S4 yellow and orange blocks of data). Thus, it is unclear if there is a TLR2-dependent mechanism of IRF induction. During our previous study^12^, the supplier had altered the amino acid sequence of the AS and AS2 proteins. Specifically, they made the following changes: F817P, A892P, A899P, A942P, K986P, and V987P. We reported that the mutated versions of AS and AS2 do not induce robust increases in CXCL10 protein levels^12^. In the present study we found that the mutated AS and AS2 did not activate IRF (Fig. 7c), and thus in this sense, the CXCL10 protein data correlates with IRF activation.

Our studies with PBMCs clearly revealed that both AS and AS2 can induce CXCL10 production and that neither heat-treated AS and AS2 nor proline mutants of AS and AS2 induce CXCL10 (Fig. 8a). S2 from RayBiotech (RS2) did not induce CXCL10 production by PBMCs but did induce production by THP-1 macrophages (Fig. 8b). The difference between the response of PBMCs (no response) and THP-1 macrophages (response) to RS2 treatment could be due to PBMCs not being differentiated. The difference in the response of PBMCs to treatment with AS2 (response) and RS2 (no response) could be due to slight differences in the amino acid sequences of AS2 and RS2 (see comparison in methods section). Interestingly, S2 supplied by Biotechne (BS2), did not induce CXCL10 production by either PBMCs or THP-1 macrophages (Fig. 8b). In previous studies utilizing commercially available proteins, including spike proteins, inconsistency across suppliers, such as seen with BS2 versus RS2, AS2, and AS (Fig. 8), has raised the concern that the induction is due to the protein samples being contaminated with LPS^19,20,27^.

In this regard, on the one hand, BS2 had a lower company-reported LPS level compared to AS and AS2 and did not induce CXCL10 production (Fig. 8b) which supports the conjecture that contaminating LPS plays a role in CXCL10 induction. On the other hand, there are numerous pieces of evidence suggesting potential contaminating LPS is not involved including the following. First, polymyxin B (PB), which neutralizes the activity of LPS^28^, has been used by others to “rule out” or “rule in” a role for possible LPS contamination in cytokine induction^19,20,28^. In the current study, all of the spike protein induction experiments were conducted in the presence of PB and induction of CXCL10 was clearly observed (Figs. 1, 2, 4, 8). Further, we previously reported that addition of PB did not affect AS and AS2 induced production of CXCL10 by THP-1 macrophages^12^. Note that PB clearly ablates LPS induction of CXCL10 production by THP-1 macrophages (Fig. 8c and^12^). We also preincubated BS2 with LPS and treated THP-1 macrophages with this solution in the presence or absence of PB (Fig. 8d). We found that the solution without PB induced CXCL10 production and that PB readily inhibited this induction, suggesting that the activity of any contaminating LPS-spike protein adduct would be inhibited by PB (Fig. 8d). Second, we have previously reported that AS and AS2, but not LPS, induce CXCL10 production by THP-1 macrophages in the presence of a TLR4 (a key LPS PRR) antagonist LPS-RS^12^. Third, we found that neither AS nor AS2 induce p65 phosphorylation at serine 536 (Fig. 6c,d) which is a known consequence of LPS treatment^30,31^ as seen in Fig. 6c. Fourth, we observed that AS and AS2 (Fig. 8a), but not LPS (Fig. 8c), induced CXCL10 production by PBMCs indicating that any potential LPS contamination in the AS and AS2 preparations would not cause induction. Fifth, we reported previously^12^ that AS1 and AS2 gave a similar positive result in the LAL LPS detection assay in the *absence* of PB yet AS1 did not induce CXCL10 production in the *absence* of PB while AS2 did induce illustrating a lack of correlation between LAL results and CXCL10 induction. The literature provides a sixth point. Specifically, Petruk et al.^32^ found that 8-h treatment of PBMCs with AS induced cytokine (e.g. IFN-β) production. The reported level of LPS in the protein preparation they used was quite low (30 fg/μg protein) and they concluded that AS itself induces cytokine expression^32^.

Thus, while contaminating LPS, or any other contaminant(s), cannot be entirely ruled out, the evidence in totality indicates that (i) a simple explanation that contaminating LPS underlies observed AS/AS2/RS2 induced THP-1 and PBMC CXCL10 production is not consistent with all of the data and (ii) the S and S2 spike proteins themselves can induce cytokine expression. Quite interestingly, this entire issue is further complicated by the fact that, Petruk et al.^32^ have provided evidence that the spike protein binds LPS and have suggested that in a pathophysiological setting LPS bound to the spike protein may cause a marked cytokine mediated inflammatory response. Thus, the best in vitro model in the end may be the spike protein with a small amount of LPS added in to simulate certain pathophysiological conditions.

Finally, in probing S and S2 induction of CXCL10 or cytokines in general, it is important to consider the following conjectures that have yet to be thoroughly investigated and may or may not prove to be relevant: (i) the recombinant proteins discussed herein and used by others have small differences in their amino acid sequences (see methods section that details these differences) which could influence their ability to induce cytokine expression; (ii) the disparate findings for the spike proteins between the EndoLISA results in the presence of PB (essentially zero) and LAL results in the presence of PB (non-zero) that we reported previously^12^ may suggest that the spike proteins themselves contribute to a positive signal in the LAL assay-an assay which is known to frequently yield false-positives^33^; (iii) PBMCs can be differentiated into different macrophage phenotypes each of which may have varying responses to spike proteins; and (iv) the spike protein of the SARS-CoV-2 has been and continues to evolve and any mutations could affect the ability of the spike protein to induce cytokine expression.

## Methods

### Cell culture

The THP-1 human monocyte cell line (TIB-202, ATCC; Manassas, Virginia), dual reporter THP-1 cells (thpd-nfis, InvivoGen; San Diego, CA; referred to herein as DRTHP1) and a *TLR2* knockout version of the dual cells (thpd-kotlr2, InvivoGen; referred to herein as KO-DRTHP1) were cultured according to the manufacturers’ protocols. In brief, THP-1 cells were cultured in RPMI-1640 medium (302,001, ATCC; Manassas, Virginia) supplemented with 10% FBS (35015CV, Corning; Corning, New York), 0.05 mM of 2-mercaptoethanol (Millipore, Billerica, MA), and 5% penicillin/streptomycin (P4333-100ML, Sigma-Aldrich; St. Louis, MO), at 37 °C with 5.0% CO_2_. The same media was used for the DRTHP1 and KO-DRTHP1 cells except the media was supplemented with 100 μg/ml Normocin. 10 µg/ml of blasticidin and 100 μg/ml of Zeocin™ were added to the media every other passage to maintain selection. To differentiate THP-1, DRTHP1 and KO-DRTHP1 monocytes to a macrophage phenotype, the cells were treated with 50 ng/mL of phorbol 12-myristate 13-acetate (PMA) (5244001MG, MilliporeSigma; Burlington, VT) in growth media for 48 h (THP-1 cells) and three hours (DRTHP1 and KO-DRTHP1 cells). Subsequently, the adherent differentiated cells were washed with RPMI-1640 medium and maintained in growth media (no PMA) at 37 °C and 5.0% CO_2_. The cells were ready for treatment at day four post-PMA treatment. We refer to the differentiated THP-1 monocytes as THP-1 macrophages throughout the paper.

### PBMC preparation

Peripheral blood mononuclear cells (PBMCs) were obtained from ImmuoSpot (Shaker Heights, OH; cat# CTL-UP1). The cells were thawed and resuspended according to the ImmnoSpot standard protocol. In brief, thawed PBMCs were resuspended in anti-aggregate medium ImmunoSpot ( Cat# CTL-AA-010) and washed twice in anti-aggregate medium. The final pellet was resuspended to 4 × 10^6^ PBMCs/ml in CTL-Test™ Medium ImmunoSpot (Cat# CTLT-010) supplemented with L-glutamine (1 vol %). Recovered PBMCs were used immediately in the experiments.

### Treatment of cells with SARS-CoV-2 proteins, Pam3CSK4, SC-514 and LPS

We obtained the SARS-CoV-2 proteins from ACROBiosystems (Delaware Technology Park; Newark, DE). Specifically, we obtained the extracellular domain of SARS-CoV-2 full length S protein (S) (cat# SPN-C52H8; Val 16-Pro 1213), mutated S (cat# SPN-C52H9; Val 16-Pro 1213), the S2 subunit (S2) (cat# S2N–C52H5; Ser 686-Pro 1213), or mutated S2 (cat# S2N–C52H5; lot# P3840b-2087F1-T7; Ser 686-Pro 1213). The mutated AS and AS2 proteins had the following substitutions: F817P, A892P, A899P, A942P, K986P, and V987P. Both versions of the AS protein had the following substitutions R683A and R685A. Note that the catalog number for S2 was not changed by ACROBiosystems when the proline substitutions were introduced. We also obtained the S2 protein from RayBitoech (Peachtree Corners; GA; cat# 230–30,613; Met 697-Pro 1213) and Biotechne (Minneapolis, MN; cat# 10,594-CV; Ser 686-Lys 1211). All proteins were expressed in human embryonic kidney 293 (HEK293) cells and contained a poly-His Tag. To distinguish between the various sources of spike protein, a letter was added in front of S and S2 to indicate the supplier: AS, AS2 indicate spike protein supplied by ACROBiosystems; RS2, BS2 indicate S2 supplied by RayBiotech and Biotechne, respectively. Unless otherwise noted, all spike protein treatments and media controls were done in growth media containing 30 µg/mL polymyxin B (PB) (92,283–10 ML, Sigma-Aldrich; St. Louis, MO) which inhibits potential contaminating LPS activity^28^. Communication with ACROBiosystems revealed that the AS protein exists predominantly as a trimer. To estimate the concentration of trimeric AS and mutated AS used, divide the values reported in the figures for these two proteins by 3. Heat treatment of spike proteins was at 95 ℃ for 30 min. Pam3CSK4 (tlrl-pms, InvivoGen) was used as a positive control inducer of NF-κB and SC-514 (Santa Cruz Biotechnology; Santa Cruz CA; cat#SC-205504) was used to probe the role of NF-κB. LPS (InvivoGen; San Diego, CA; cat# tlrl-3pelps) was used at a nominal 1 EU/ml.

### Proteome profiler and ELISA quantification of cytokine proteins

The proteome profiler human cytokine array kit (a membrane based-sandwich immunoassay; ARY005B, R&D Systems; Minneapolis, MN) was used to quantify the presence of proinflammatory cytokines in culture supernatants according to the manufacturer’s protocol^23^. In brief, the blots were exposed to the macrophage supernatants that were diluted 1:7.5 with the assay diluent buffer. Subsequently, the blots were washed and the Bio-Rad ChemiDoc XRS + Molecular Imager (Hercules, CA) was used to develop the images shown. The human CXCL10/IP-10 ELISA kit (KAC2361, ThermoFisher; Waltham, MA) was used to quantify the presence of CXCL10 protein in culture supernatants. The assay was performed following the manufacturer's protocol^12^. CXCL10 concentrations were estimated from a standard curve generated from the standards provided with the kit.

### Reverse transcription quantitative real-time polymerase chain reaction (RT-qPCR)

The THP-1 macrophages were treated with 25 nM of S and S2 proteins alone or in combination with 25 µM COB-187 or 1% DMSO (carrier control for COB-187). After a 6-h incubation, the THP1 macrophages were harvested, and the mRNA was isolated via RNeasy Mini Kit (74,104, Qiagen; Germantown, MD) according to the manufacturer’s protocol. The mRNA was also isolated from non-treated THP-1, DRTHP1, and KO-DRTHP1 macrophages to determine if they express *TLR2*. Preparation of cDNA was achieved using the high-capacity cDNA reverse transcription kit with RNase Inhibitor (Applied Biosystems/ThermoFisher). SYBR Green biochemistry [iTaq Universal SYBR Green Supermix (1,725,121), Bio-Rad Laboratories, Inc.; Hercules, CA] was used to perform qPCR to quantify gene expression. Taqman Gene Expression Assays that were used as qPCR primers to determine the levels of *CXCL10* and *TLR2* gene expression include: *CXCL10* (Hs00171042_m1, ThermoFisher) and *TLR2* (Hs00610101_m1, ThermoFisher). The CFX96 Touch Real-Time PCR Detection System (Bio Rad; Hercules, CA) was used to perform the qPCR. Housekeeping genes for *CXCL10* evaluation were *Actinβ* (Hs99999903_m1, ThermoFisher), and *HPRT1* (Hs02800695_m1, ThermoFisher) and for *TLR2* were *Actinβ* and *GAPDH*​ (Hs99999905_m1, ThermoFisher). The ΔΔCT method was used to determine the level of expression of *CXCL10* versus media control.

### Flow cytometric analysis of TLR2 protein expression

Flow cytometric analysis was used to qualitatively determine the level of expression of TLR2 on the cell surface of THP-1, DRTHP1 and KO-DRTHP1 macrophages. In brief, the macrophages were washed with Dulbecco’s phosphate-buffered saline without Ca^2+^ and Mg^2+^ (DPBS) and then detached from the surface of the flask using Gibco™ cell dissociation buffer, enzyme-free, Hanks' balanced salt solution (13,150,016, Gibco; Gaithersburg, MD). The harvested cells were washed with DPBS with Ca^2+^ and Mg^2+^ (DPBS + ; ThermoFisher) containing 2% FBS. Suspensions of 2 × 10^5^ macrophages were treated with 20 µg/mL Fc receptor binding inhibitor polyclonal antibody, functional grade, eBioscience™ (16–9161-73, ThermoFisher), for 15 min on ice in the dark to inhibit the antibodies from binding to the Fc receptors. The macrophages were then treated with 10 µg/mL CD282 (TLR2) monoclonal antibody (mAb) (14–9922-82, ThermoFisher) or 20 µg/mL mouse IgG2a kappa isotype control (14–4724-82, ThermoFisher) for 40 min in the dark on ice. Subsequently, the treated macrophages were washed twice with DPBS + /2% FBS and then stained with 20 µg/mL PE labeled goat anti-mouse IgG2a for 30 min (ThermoFisher, 31,863). The stained macrophages were washed three times with DPBS + /2% FBS and once with DPBS. The macrophages were then centrifuged and resuspended in 200 µL DPBS. A FACS Aria Special Order Research Product flow cytometer/cell sorter (BD Biosciences, Franklin Lakes, New Jersey) was used to analyze the cells and FlowJo software (FlowJo LLC, Ashland, Oregon) was used to analyze the data.

### Determination of activation of NF-κB and IRF

To determine if spike proteins activate NF-κB or IRF transcription factors, DRTHP1 and KO-DRTHP1 macrophages were treated with 25 nM of AS and AS2 proteins. As a positive control for NF-κB activation, the macrophages were treated with 1000 ng/mL Pam3CSK4 for 6 hours^26^. Subsequently, the supernatants were harvested and analyzed to determine if NF-κB and/or IRF were activated. The QUANTI Blue assay (rep-qbs, InvivoGen; San Diego, CA) was used to determine the level of secreted alkaline phosphatase (SEAP) reporter protein in the macrophage supernatants which correlates with NF-κB activation. The assay was performed according to the manufacturer’s protocol. Briefly, 180 μL aliquots of QUANTI-Blue solution and 20 μL of macrophage supernatants from the various treatments were mixed in wells of a flat‑bottom 96-well plate. The plate was then incubated for 2 h at 37 ℃. Subsequently, the 620 nm optical density of the solutions in each well was determined. The optical density correlates with the level of SEAP in the solution and thus the level of NF-κB activation. The QUANTI-Luc™ assay (rep-qlc1, InvivoGen; San Diego, CA) was used to determine the level of Lucia luciferase reporter protein in the macrophage supernatants which correlates with the level of IRF activation. The assay was performed according to the manufacturer’s protocol. Briefly, 20 µl of macrophage supernatants from the various treatment groups were dispensed into wells of a 96-well white (opaque) plate. Subsequently, 50 µl of QUANTI-Luc™ assay solution was added to each well. The luminescence of the solution in each well was then determined immediately using a luminometer (Synergy™ H1M, BioTek Instruments, Inc, VT). The luminescence correlates with the level of Lucia luciferase in the solution and thus the level of IRF activation.

### Western blot analysis of NF-κB

Total protein was isolated from THP-1 macrophages using a standard lysis buffer (10 mM Tris HCl, pH 7.5, 150 mM NaCl, 1% NP-40) containing protease inhibitors followed by sonication. For the detection of phosphorylated NF-ĸB/p65 (Serine 536), total NF-ĸB/p65, and β-Actin, 30 µg of total protein isolated from the THP-1 macrophages at the indicated time points was subjected to SDS-PAGE and western blot analysis using an antibody specific for the detection of phospho NF-ĸB (Serine 536), NF-ĸB, and β-Actin (3031S, 8242S, and 4967S, respectively, Cell Signaling Technologies, Danvers, MA, USA) at a dilution of 1:500, 1:500, and 1:1000, respectively. Blots were first probed with phospho NF-ĸB (Serine 536), then stripped and re-probed with an antibody specific for the detection of total NF-ĸB and/or β-Actin. Goat anti-rabbit IgG-HRP conjugated secondary antibody (Invitrogen, Thermo Fisher Scientific, Waltham, MA, USA) at a dilution of 1:20,000 was used. For visualization of signals, the ECL Western Blotting Analysis System (Amersham/Cytiva Life Sciences, Marlborough, MA, USA) was used. Western blots were read using the ChemiDoc MP Imaging System (BioRad Laboratories, Hercules, CA, USA).

### Statistics

To determine if observed differences between treatment groups were statistically significant, a one-sample or a two-sample, two-tailed, unpaired Student’s t-test was used. All statistical analyses were performed using the GraphPad Prism 8.3.2 (GraphPad Software Inc., San Diego, CA) or the Statology online t-test calculator. Multiple comparisons were evaluated using a Bonferroni correction wherein the p value for significance was divided by the number of comparisons, e.g., for 2 comparisons, *P* < 0.025 was considered significant.

### Supplementary Information

Supplementary Information.

## Data Availability

The datasets generated during the current study are available from the corresponding author on reasonable request.
