# Supplementary material for: Mechanistic insights into SARS-CoV-2 spike protein induction of the chemokine CXCL10
Source: Sci Rep. 2024 May 16;14:11179. doi: 10.1038/s41598-024-61906-6 (PMC11096305; doi:10.1038/s41598-024-61906-6)
Supplement: Supplementary file 1 — Supplementary Information. [file 41598_2024_61906_MOESM1_ESM.pdf]

Supplementary Information

Mechanistic Insights into SARS-CoV-2 Spike Protein Induction of the Chemokine CXCL10

Davoud Ghazanfari[a], Maria Cecilia Courreges[b], Lydia E. Belinski[a,c], Michael J. Hogrell[a,c], Jacob Lloyd[a], Stephen C. Bergmeier[c,d], Kelly D. McCall[b,c,e,f,g,h], *Douglas J. Goetz[a,c]

[a]Department of Chemical and Biomolecular Engineering, Ohio University, Athens, OH 45701, USA

[b]Department of Specialty Medicine, Ohio University, Athens, OH 45701, USA

[c]Biomedical Engineering Program, Ohio University, Athens, OH 45701, USA

[d]Department of Chemistry and Biochemistry, Ohio University, Athens, OH 45701, USA

[e]The Diabetes Institute, Ohio University, Athens, OH 45701, USA

[f]Molecular and Cellular Biology Program, Ohio University College of Arts & Sciences, Athens OH 45701, USA

[g]Department of Biological Sciences, Ohio University College of Arts & Sciences, Athens, OH, 45701, USA

[h]Department of Biomedical Sciences, Ohio University Heritage College of Osteopathic Medicine, Athens, OH 45701, USA

*Corresponding author: Douglas J. Goetz[a,c]

Email: goetzd@ohio.edu

Ghazanfari et al.
Supplementary Figure S1.

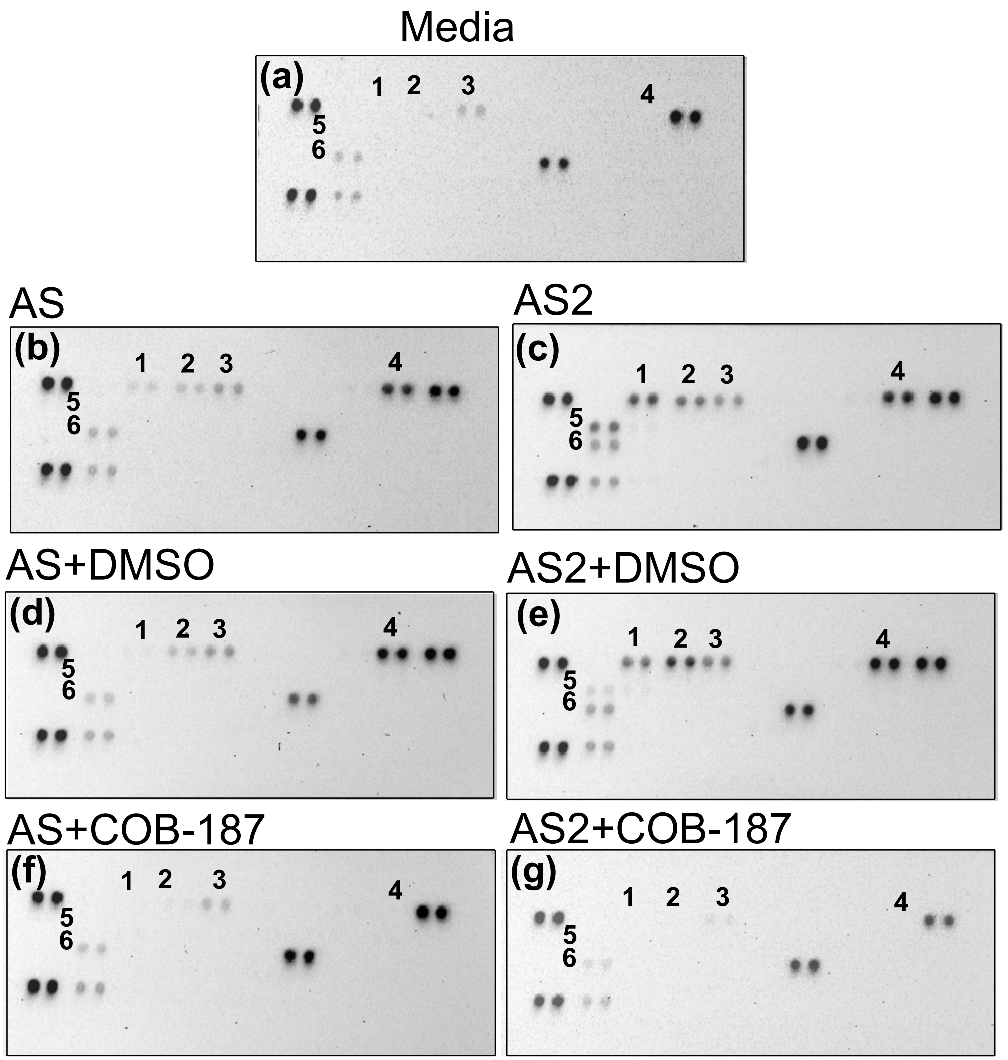

Map of proteins detected

Coordinate	**Target**
1	CCL2/MCP-1
2	CCL3/CCL4/MIP-1alpha/beta
3	CCL5/RANTES
4	CXCL10/IP-10
5	CXCL11/I-TAC
6	IL-1ra/IL-1F3

Figure S1. S and S2 treatment increase CXCL10 protein levels and this increase is attenuated by COB-187. This figure is a replicate experiment of the representative figure presented in Fig. 1 of the main text. Protein arrays exposed to supernatants from THP-1 macrophages treated with AS and AS2 proteins +/- COB-187 revealed: supernatants from untreated THP-1 macrophages had little, if any, CXCL10 (location 4, panel a); AS or AS2 protein alone and in the presence of 1% DMSO (carrier control for COB-187) increased CXCL10 protein levels (location 4 panels b,c,d,e); COB-187 appeared to abolish the AS- and AS2-induced increase in CXCL10 protein levels (location 4 panels f,g). Note that AS2 appeared to increase levels of 3 other proteins (locations 1,2,3; panels c) that were dramatically reduced by COB-187 (locations 1,2,3; panel g) but unaffected by 1% DMSO (locations 1,2,3; panel e). The map provides the correspondence between the number on the array and the cytokine present at that location. Each cytokine is in duplicate (two dots per location). The two dark pairs of dots on the left side of each array and the dark pair of dots on the right side of the array are positive controls.

Ghazanfari et al.
Supplementary Figure S2.

Figure S2. THP-1 and DRTHP1, but not KO-DRTHP1, macrophages have surface expressed TLR2 protein. Further replicates of the representative data presented in Figs. 3 of the main text. Flow cytometric analysis revealed that a mAb to TLR2 bound to THP-1 **(a,b)** and DRTHP1 **(c)** macrophages (red histograms) to a greater extent than an isotype matched control (blue histograms) suggesting that these cells express surface TLR2 protein. **(d)** Flow cytometric analysis revealed that a mAb to TLR2 did not bind to KO-DRTHP1 macrophages (red histogram) to a greater extent than an isotype matched control (blue histogram) suggesting that KO-DRTHP1 macrophages do not express surface TLR2 protein.

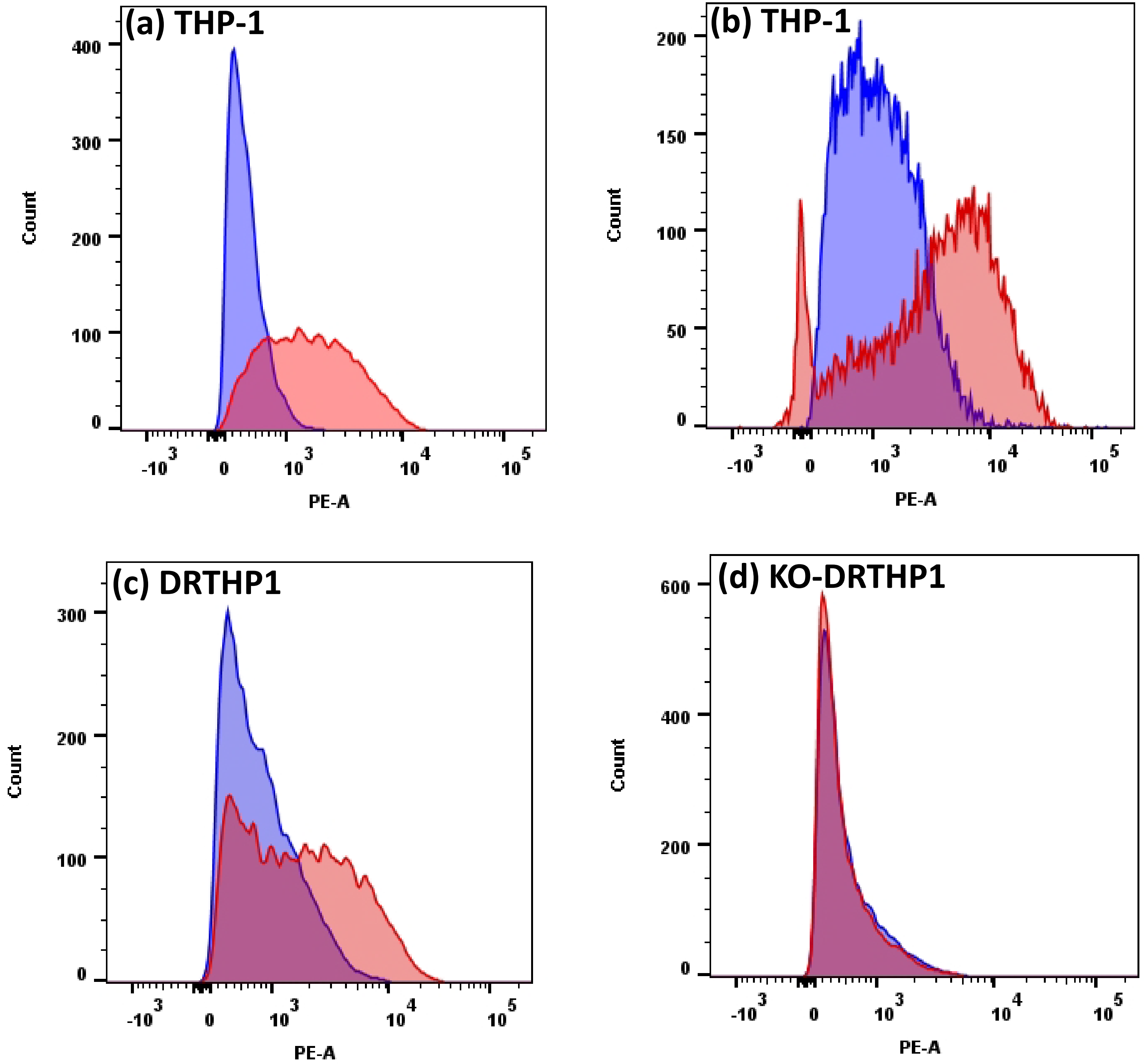

Ghazanfari et al.
Supplementary Figure S3.

Figure S3. AS and AS2 do not appear to activate NF-κB in THP-1 macrophages. The following two pages contain the original blots from which Figs. 6c and 6d were generated.

Fig. 6c. Same blot, stripped and re-probed for total NFkB-p65 and β-Actin

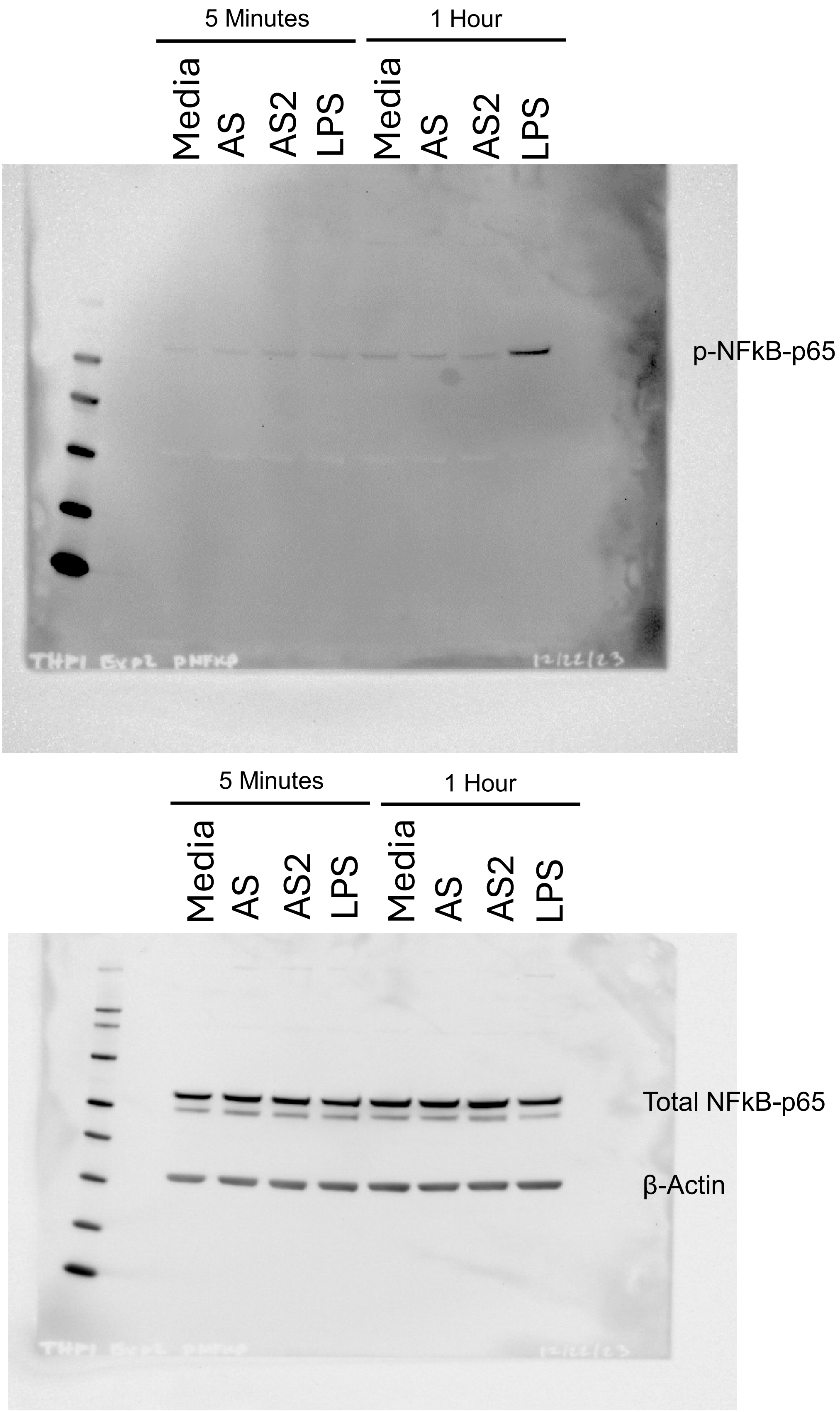

Fig. 6d. Same blot, stripped and re-probed for total NFkB-p65 and β-Actin

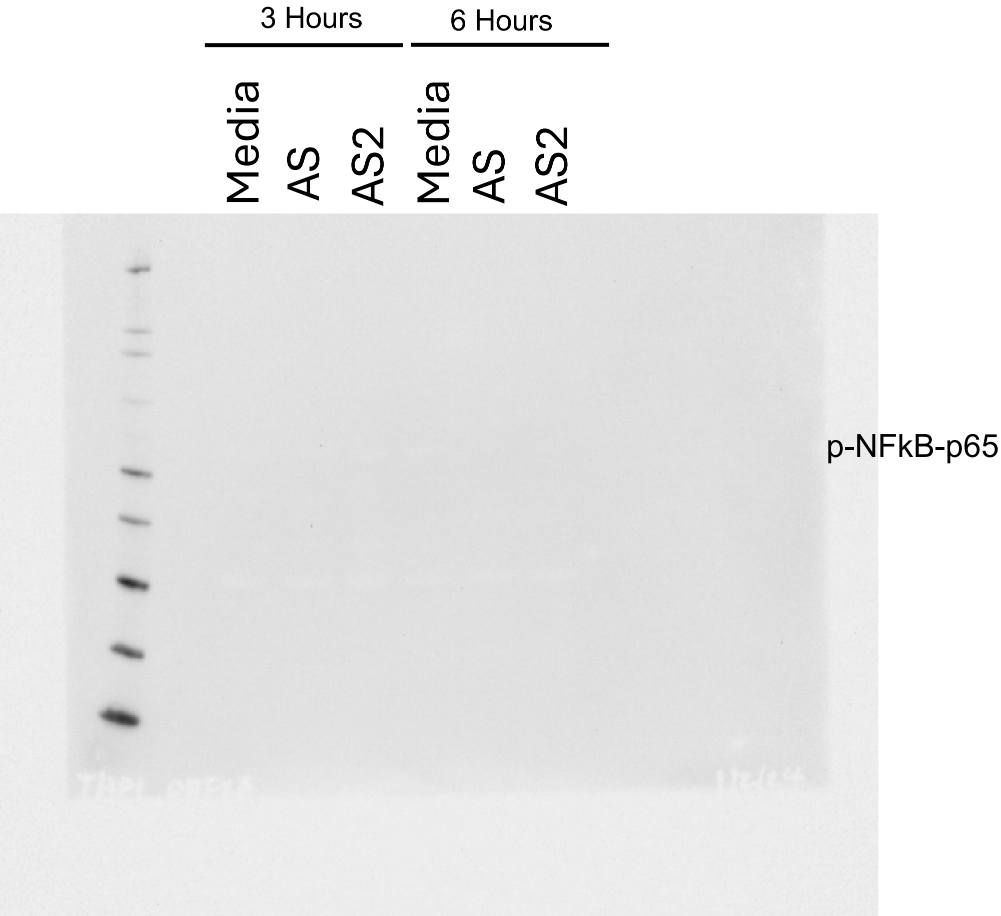

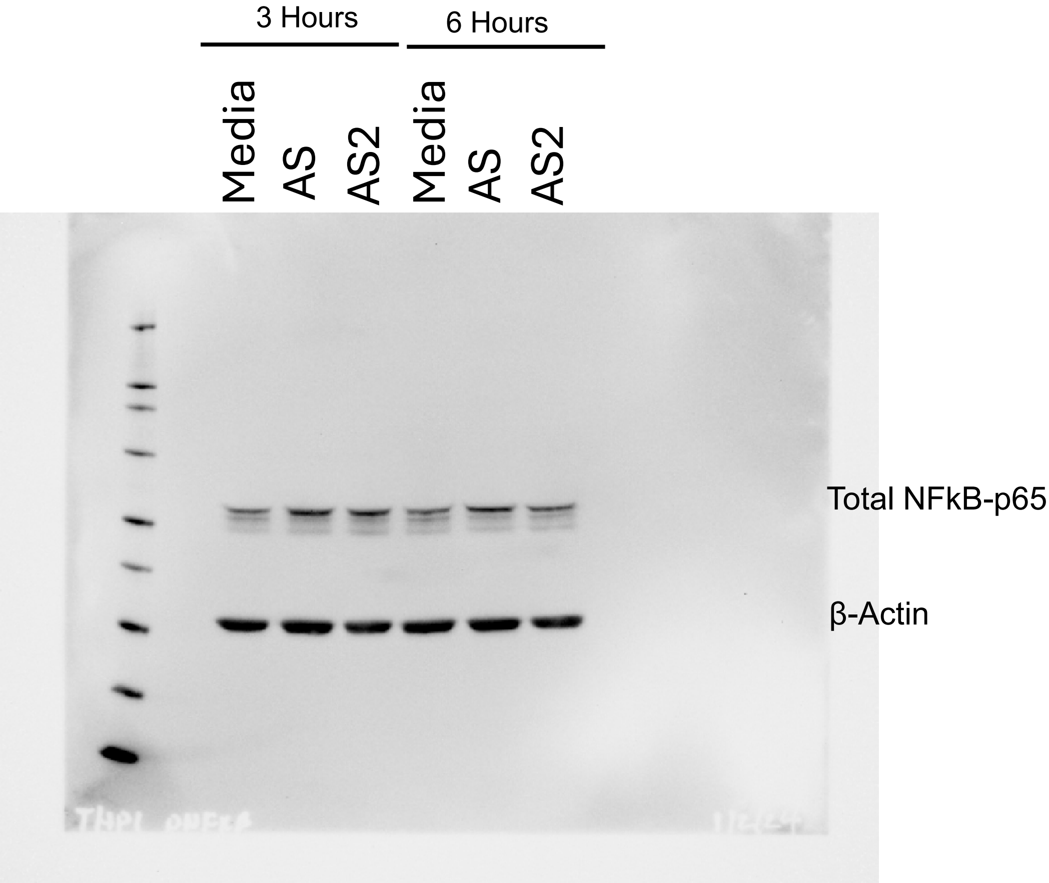

Supplementary Table S1. Replicate experiments for Fig. 4. Mean and SD determined from duplicates for each condition within a replicate. Compared to media control via t-test: ∗p <0.025; ∗∗p< 0.01; and ***p<0.001. Threshold for significant difference is p<0.05 and p<0.025 without and with Bonferroni correction, respectively. AS and AS2 were at 25 nM. Replicate 1 from each set is presented in Fig. 4.

Figure	Rep	Treatment	Cell Type	Assay	Mean	SD	Significance Without Correction	Significance with Bonferroni Correction
4a	1	Media	DRTHP1	ELISA	0.12	0.020	-	-
4a	1	AS	DRTHP1	ELISA	2.01	0.244	**	**
4a	1	AS2	DRTHP1	ELISA	2.07	0.216	**	**
4a	2	Media	DRTHP1	ELISA	0.04	0.004	-	-
4a	2	AS	DRTHP1	ELISA	0.52	0.012	***	***
4a	2	AS2	DRTHP1	ELISA	0.51	0.018	***	***
4a	3	Media	DRTHP1	ELISA	0.14	0.008	-	-
4a	3	AS	DRTHP1	ELISA	2.36	0.040	***	***
4a	3	AS2	DRTHP1	ELISA	2.41	0.099	***	***
4b	1	Media	KO-DRTHP1	ELISA	0.13	0.005	-	-
4b	1	AS	KO-DRTHP1	ELISA	1.83	0.271	*	*
4b	1	AS2	KO-DRTHP1	ELISA	2.18	0.132	**	**
4b	2	Media	KO-DRTHP1	ELISA	0.04	0.000	-	-
4b	2	AS	KO-DRTHP1	ELISA	0.44	0.022	**	**
4b	2	AS2	KO-DRTHP1	ELISA	0.50	0.011	***	***
4b	3	Media	KO-DRTHP1	ELISA	0.14	0.003	-	-
4b	3	AS	KO-DRTHP1	ELISA	2.25	0.107	**	**
4b	3	AS2	KO-DRTHP1	ELISA	2.50	0.052	***	***

Supplementary Table S2. Replicate experiments for Fig. 5. Mean and SD were determined from duplicates for each condition within each replicate. Compared to media control via t-test: NS indicates not significant; LTC indicates lower than control; #p<0.05; **p< 0.01; and ***p<0.001. Threshold for significant difference is p<0.05 and p<0.025 without and with Bonferroni correction, respetively. A Bonferroni correction is not needed for Figs. 5c and 5d since only a single comparison was made. AS and AS2 were at 25 nM. Pam3CSK4 was at 1000 ng/ml. Replicate 1 from each set is presented in Fig. 5.

Figure	Rep	Treatment	Cell Type	Assay	Mean	SD	Significance without Correction	Significance with Bonferroni Correction
5a	1	Media	DRTHP1	NF-kB	1.21	0.059	-	-
5a	1	AS	DRTHP1	NF-kB	1.25	0.021	NS	NS
5a	1	AS2	DRTHP1	NF-kB	1.52	0.200	NS	NS
5a	2	Media	DRTHP1	NF-kB	1.78	0.025	-	-
5a	2	AS	DRTHP1	NF-kB	1.86	0.108	NS	NS
5a	2	AS2	DRTHP1	NF-kB	1.87	0.121	NS	NS
5a	3	Media	DRTHP1	NF-kB	1.33	0.015	-	-
5a	3	AS	DRTHP1	NF-kB	1.39	0.141	NS	NS
5a	3	AS2	DRTHP1	NF-kB	1.21	0.095	NS	NS
5a	4	Media	DRTHP1	NF-kB	1.88	0.003	-	-
5a	4	AS	DRTHP1	NF-kB	1.66	0.022	**LTC	**LTC
5a	4	AS2	DRTHP1	NF-kB	1.76	0.039	#LTC	NS
5a	5	Media	DRTHP1	NF-kB	1.10	0.008	-	-
5a	5	AS	DRTHP1	NF-kB	1.10	0.006	NS	NS
5a	5	AS2	DRTHP1	NF-kB	1.16	0.029	NS	NS
5a	6	Media	DRTHP1	NF-kB	1.81	0.037	-	-
5a	6	AS	DRTHP1	NF-kB	1.84	0.045	NS	NS
5a	6	AS2	DRTHP1	NF-kB	2.00	0.158	NS	NS
5b	1	Media	KO-DRTHP1	NF-kB	1.11	0.083	-	-
5b	1	AS	KO-DRTHP1	NF-kB	0.88	0.095	NS	NS
5b	1	AS2	KO-DRTHP1	NF-kB	1.07	0.025	NS	NS
5b	2	Media	KO-DRTHP1	NF-kB	1.93	0.056	-	-
5b	2	AS	KO-DRTHP1	NF-kB	2.08	0.259	NS	NS
5b	2	AS2	KO-DRTHP1	NF-kB	1.83	1.835	NS	NS
5b	3	Media	KO-DRTHP1	NF-kB	0.85	0.009	-	-
5b	3	AS	KO-DRTHP1	NF-kB	0.96	0.126	NS	NS
5b	3	AS2	KO-DRTHP1	NF-kB	0.78	0.071	NS	NS
5c	1	Media	DRTHP1	NF-kB	1.11	0.003	-	-
5c	1	Pam3CSK4	DRTHP1	NF-kB	1.91	0.064	**	-
5c	2	Media	DRTHP1	NF-kB	1.85	0.004	-	-
5c	2	Pam3CSK4	DRTHP1	NF-kB	2.55	0.066	**	-
5c	3	Media	DRTHP1	NF-kB	1.71	0.017	-	-
5c	3	Pam3CSK4	DRTHP1	NF-kB	2.41	0.019	***	-
5d	1	Media	KO-DRTHP1	NF-kB	1.17	0.188	-	-
5d	1	Pam3CSK4	KO-DRTHP1	NF-kB	1.13	0.011	NS	-
5d	2	Media	KO-DRTHP1	NF-kB	1.82	0.005	-	-
5d	2	Pam3CSK4	KO-DRTHP1	NF-kB	1.95	0.206	NS	-

Supplementary Table S3. Replicate experiments for Fig. 6 a,b. Mean and SD determined from duplicates for each condition within a replicate. For 6a: Compared to DMSO via two sample t-test. For 6b, compared to 100% via one sample t-test. NS indicates not significant; #p<0.05. Threshold for significant difference is p<0.05. A Bonferroni correction was not used for the one sample t-test in 6b. AS and AS2 were at 25 nM. Replicate 1 is presented in Fig. 6a.

Figure	Rep	Treatment	Cell Type	Assay	Mean	SD	Significance Without Correction	Significance with Bonferroni Correction
6a	1	AS + DMSO	WT THP-1	ELISA	3.02	0.037	-	-
6a	1	AS + SC-514	WT THP-1	ELISA	2.13	0.495	NS	NS
6a	1	AS2 + DMSO	WT THP-1	ELISA	2.59	0.448	-	-
6a	1	AS2 + SC-514	WT THP-1	ELISA	2.28	0.387	NS	NS
6a	2	AS + DMSO	WT THP-1	ELISA	1.66	0.284	-	-
6a	2	AS + SC-514	WT THP-1	ELISA	1.28	0.123	NS	NS
6a	2	AS2 + DMSO	WT THP-1	ELISA	1.75	0.038	-	-
6a	2	AS2 + SC-514	WT THP-1	ELISA	1.87	0.019	NS	NS
6a	3	AS + DMSO	WT THP-1	ELISA	1.70	0.409	-	-
6a	3	AS + SC-514	WT THP-1	ELISA	1.09	0.096	NS	NS
6a	3	AS2 + DMSO	WT THP-1	ELISA	2.49	0.153	-	-
6a	3	AS2 + SC-514	WT THP-1	ELISA	2.66	0.079	NS	NS

Figure	Rep	Treatment	Cell Type	Assay	% relative to DMSO control	SD	Significance Without Correction	Significance with Bonferroni Correction
6b	1	AS + SC-514	WT THP-1	ELISA	70.4	-	-	-
6b	1	AS2 + SC-514	WT THP-1	ELISA	88.2	-	-	-
6b	2	AS + SC-514	WT THP-1	ELISA	77.2	-	-	-
6b	2	AS2 + SC-514	WT THP-1	ELISA	107.0	-	-	-
6b	3	AS + SC-514	WT THP-1	ELISA	64.5	-	-	-
6b	3	AS2 + SC-514	WT THP-1	ELISA	106.8	-	-	-
6b	Ave	AS + SC-514	WT THP-1	ELISA	70.7	6.37	#	-
6b	Ave	AS2 + SC-514	WT THP-1	ELISA	100.7	10.80	NS	-

Supplementary Table S4. Replicate experiments for Fig. 7. Mean and SD were determined from duplicates for each condition within each replicate. Compared to media control via t-test: NS indicates not significant; LTC indicates lower than control: #p<0.05; *p<0.025; **p< 0.01; %p<0.0083 (the cutoff for significance with a Bonferroni correction involving 6 comparisons); and ***p<0.001. Threshold for significant difference is p<0.05 without Bonferroni correction. Threshold for significant difference is p<0.025 (Figs. 7a,b) and p<0.0083 (Fig. 7c) with Bonferroni correction. AS and AS2 were at 25 nM. Replicate 1 from each set is presented in Fig. 7. Note that the positive controls (AS, AS2) for the replicates in Fig. 7c provide two additional replicates for 7a.

Figure	Rep	Treatment	Cell Type	Assay	Mean	SD	Significance without Correction	Significance with Bonferroni Correction
7a	1	Media	DRTHP1	IRF	201	8	-	-
7a	1	AS	DRTHP1	IRF	1219	155	*	*
7a	1	AS2	DRTHP1	IRF	1922	296	*	*
7a	2	Media	DRTHP1	IRF	429	131	-	-
7a	2	AS	DRTHP1	IRF	2918	145	**	**
7a	2	AS2	DRTHP1	IRF	6065	354	**	**
7a	3	Media	DRTHP1	IRF	489	14	-	-
7a	3	AS	DRTHP1	IRF	2351	372	*	*
7a	3	AS2	DRTHP1	IRF	11835	440	***	***
7a	4	Media	DRTHP1	IRF	403	25	-	-
7a	4	AS	DRTHP1	IRF	2772	199	**	**
7a	4	AS2	DRTHP1	IRF	1218	48	**	**
7b	1	Media	KO-DRTHP1	IRF	344	8	-	-
7b	1	AS	KO-DRTHP1	IRF	604	28	**	**
7b	1	AS2	KO-DRTHP1	IRF	2229	108	**	**
7b	2	Media	KO-DRTHP1	IRF	777	125	-	-
7b	2	AS	KO-DRTHP1	IRF	1969	255	#	NS
7b	2	AS2	KO-DRTHP1	IRF	4025	1301	NS	NS
7b	3	Media	KO-DRTHP1	IRF	562	13	-	-
7b	3	AS	KO-DRTHP1	IRF	3974	40	***	***
7b	3	AS2	KO-DRTHP1	IRF	9327	294	***	***
7c	1	Media	DRTHP1	IRF	764	27	-	-
7c	1	AS	DRTHP1	IRF	4364	24	***	***
7c	1	Heat treat AS	DRTHP1	IRF	477	43	*LTC	NS
7c	1	Mutated AS	DRTHP1	IRF	659	15	*LTC	NS
7c	1	AS2	DRTHP1	IRF	10120	545	%	%
7c	1	Heat treat AS2	DRTHP1	IRF	747	1	NS	NS
7c	1	Mutated AS2	DRTHP1	IRF	607	66	NS	NS
7c	2	Media	DRTHP1	IRF	420	33	-	-
7c	2	AS	DRTHP1	IRF	1396	98	%	%
7c	2	Heat treat AS	DRTHP1	IRF	278	71	NS	NS
7c	2	Mutated AS	DRTHP1	IRF	341	15	NS	NS
7c	2	AS2	DRTHP1	IRF	3134	169	%	%
7c	2	Heat treat AS2	DRTHP1	IRF	335	23	NS	NS
7c	2	Mutated AS2	DRTHP1	IRF	364	41	NS	NS

Supplementary Table S5. Replicate experiments for Fig. 8. Mean and SD determined from duplicates for each condition within a replicate; quadruplicates used for RS2 8b, Rep 1. Compared to media matched control for 8a, 8b, 8c via t-test; compared to same condition with PB for 8d via t-test: NS indicates not significant; NP indicates not performed; LTC indicates lower than control; #p<0.05;**p< 0.01; and ***p<0.001. Threshold for significant difference is p<0.05 without Bonferroni correction and p<0.025 with Bonferroni coretion. Concentration of spike proteins was 25 nM. Replicate 1 from each set (8a, 8b, 8c) and the 1 EU/ml condition (8d) is presented in Fig. 8.

Figure	Rep	Treatment	Cell Type	Assay	Mean	SD	Significance Without Correction	Significance with Bonferroni Correction
8a	1	AS	PBMCs	ELISA	0.84	0.070	**	**
8a	1	AS2	PBMCs	ELISA	1.31	0.059	**	**
8a	1	Media	PBMCs	ELISA	0.06	0.000	-	-
8a	1	Heat AS	PBMCs	ELISA	0.08	0.004	NP	NP
8a	1	Heat AS2	PBMCs	ELISA	0.07	0.001	NP	NP
8a	1	Mut AS	PBMCs	ELISA	0.07	0.006	NP	NP
8a	1	Mut AS2	PBMCs	ELISA	0.06	0.000	NP	NP
8a	2	AS	PBMCs	ELISA	0.63	0.057	**	**
8a	2	AS2	PBMCs	ELISA	1.20	0.039	***	***
8a	2	Media	PBMCs	ELISA	0.08	0.001	-	-
8a	2	Heat AS	PBMCs	ELISA	0.05	0.003	NP	NP
8a	2	Heat AS2	PBMCs	ELISA	0.05	0.004	NP	NP
8a	2	Mut AS	PBMCs	ELISA	0.06	0.002	NP	NP
8a	2	Mut AS2	PBMCs	ELISA	0.18	0.011	NP	NP
8b	1	RS2	WT THP-1	ELISA	1.61	0.315	**	**
8b	1	BS2	WT THP-1	ELISA	0.07	0.004	NS	NS
8b	1	Media	WT THP-1	ELISA	0.08	0.005	-	-
8b	1	RS2	PBMCs	ELISA	0.24	0.095	NS	NS
8b	1	BS2	PBMCs	ELISA	0.09	0.008	NS	NS
8b	1	Media	PBMCs	ELISA	0.10	0.007	-	-
8b	2	RS2	WT THP-1	ELISA	1.11	0.042	***	***
8b	2	BS2	WT THP-1	ELISA	0.07	0.005	NS	NS
8b	2	Media	WT THP-1	ELISA	0.08	0.002	-	-
8b	2	RS2	PBMCs	ELISA	0.24	0.196	NS	NS
8b	2	BS2	PBMCs	ELISA	0.15	0.006	**	**
8b	2	Media	PBMCs	ELISA	0.07	0.008	-	-
8c	1	LPS - PB	WT THP-1	ELISA	1.79	0.101	**	**
8c	1	LPS + PB	WT THP-1	ELISA	0.10	0.001	#LTC	NS
8c	1	Media	WT THP-1	ELISA	0.11	0.002	-	-
8c	1	Media - PB	WT THP-1	ELISA	0.07	0.002	-	-
8c	1	LPS - PB	PBMCs	ELISA	0.05	0.002	NS	NS
8c	1	LPS + PB	PBMCs	ELISA	0.05	0.001	NS	NS
8c	1	Media	PBMCs	ELISA	0.05	0.004	-	-
8c	1	Media - PB	PBMCs	ELISA	0.06	0.004	-	-
8c	2	LPS - PB	WT THP-1	ELISA	1.49	0.093	**	**
8c	2	LPS + PB	WT THP-1	ELISA	0.07	0.005	NS	NS
8c	2	Media	WT THP-1	ELISA	0.06	0.002	-	-
8c	2	Media - PB	WT THP-1	ELISA	0.07	0.007	-	-
8c	2	LPS - PB	PBMCs	ELISA	0.05	0.001	NS	NS
8c	2	LPS + PB	PBMCs	ELISA	0.05	0.000	NS	NS
8c	2	Media	PBMCs	ELISA	0.05	0.001	-	-
8c	2	Media - PB	PBMCs	ELISA	0.05	0.001		
8d	1	BS2 + 1 EU/ml LPS - PB	WT THP-1	ELISA	2.06	0.096	**	**
8d	1	BS2+1 EU /ml LPS+PB	WT THP-1	ELISA	0.11	0.001	-	-
8d	1	1 EU/ml LPS - PB	WT THP-1	ELISA	2.13	0.062	***	***
8d	1	1 EU/ml LPS + PB	WT THP-1	ELISA	0.12	0.004	-	-
8d	1	BS2	WT THP-1	ELISA	0.07	0.000	-	-
8d	1	Media - PB	WT THP-1	ELISA	0.07	0.001	-	-
8d	1	Media	WT THP-1	ELISA	0.08	0.001	-	-
8d	1	BS2 + 0.5 EU/ml LPS - PB	WT THP-1	ELISA	2.05	0.068	***	***
8d	1	BS2+0.5 EU /ml LPS+PB	WT THP-1	ELISA	0.08	0.000	-	-
8d	1	0.5 EU/ml LPS - PB	WT THP-1	ELISA	2.14	0.074	***	***
8d	1	0.5 EU/ml LPS + PB	WT THP-1	ELISA	0.12	0.000	-	-
